# A Motion Segmentation Dynamic SLAM for Indoor GNSS-Denied Environments

**DOI:** 10.3390/s25164952

**Published:** 2025-08-10

**Authors:** Yunhao Wu, Ziyao Zhang, Haifeng Chen, Jian Li

**Affiliations:** 1 College of Electronic Information and Artificial Intelligence, Shaanxi University of Science and Technology, Xi’an 710021, China; 221611034@sust.edu.cn (Y.W.); chenhaifeng@sust.edu.cn (H.C.); 2 School of Physics, Peking University, Beijing 100871, China; 1901110194@pku.edu.cn; 3 State Key Laboratory of Artificial Microstructure and Mesoscopic Physics, Beijing 100871, China

**Keywords:** simultaneous localization and mapping, optical flow, dynamic scene, semantic seg-mentation, GNSS-denied environments

## Abstract

In GNSS-deprived settings, such as indoor and underground environments, research on simultaneous localization and mapping (SLAM) technology remains a focal point. Addressing the influence of dynamic variables on positional precision and constructing a persistent map comprising solely static elements are pivotal objectives in visual SLAM for dynamic scenes. This paper introduces optical flow motion segmentation-based SLAM(OS-SLAM), a dynamic environment SLAM system that incorporates optical flow motion segmentation for enhanced robustness. Initially, a lightweight multi-scale optical flow network is developed and optimized using multi-scale feature extraction and update modules to enhance motion segmentation accuracy with rigid masks while maintaining real-time performance. Subsequently, a novel fusion approach combining the YOLO-fastest method and Rigidmask fusion is proposed to mitigate mis-segmentation errors of static backgrounds caused by non-rigid moving objects. Finally, a static dense point cloud map is generated by filtering out abnormal point clouds. OS-SLAM integrates optical flow estimation with motion segmentation to effectively reduce the impact of dynamic objects. Experimental findings from the Technical University of Munich (TUM) dataset demonstrate that the proposed method significantly outperforms ORB-SLAM3 in handling high dynamic sequences, achieving a reduction of 91.2% in absolute position error (APE) and 45.1% in relative position error (RPE) on average.

## 1. Introduction

With the proliferation of artificial intelligence-based robots [1], drones [2], and autonomous vehicles [3], SLAM has become a research hotspot in computer vision. SLAM technology enables devices to estimate their position and attitude using onboard sensors while simultaneously constructing environmental maps [4], fulfilling the localization requirements for autonomous motion.

SLAM technology is categorized into vision-based and laser-based systems depending on the sensor type used. Due to advancements in LiDAR equipment and the maturation of laser SLAM technology, it has seen widespread industrial adoption [5]. Subsequently, visual SLAM emerged as a research focus in emerging fields like robotics due to its lightweight design and cost-effectiveness [6]. Simultaneously, by leveraging color and depth data from captured images, the system demonstrates significant potential to enhance tracking efficiency and closed-loop accuracy [7].

The visual SLAM framework primarily comprises four components: visual odometry (VO), back-end optimization, loop closure, and mapping. Additionally, visual SLAM methodologies are categorized into feature-based and direct approaches. The eigenpoint method exhibits theoretical maturity and illumination robustness, but demands substantial computational resources for feature matching. PTAM [8], ORB-SLAM3 [9], and PL-SLAM [10] represent key algorithms in visual SLAM. While the direct approach eliminates feature point computation, it demonstrates reduced robustness in environments with repetitive textures or limited surface details. Recent advances in direct visual SLAM methods, such as SVO [11], DSO [12], and LSD-SLAM [13], demonstrate foundational importance in AR and UAV applications owing to their robust performance.

However, the presence of dynamic objects, such as indoor pedestrians or vehicular traffic, introduces interference to SLAM’s feature matching and position estimation processes, compromising system localization accuracy [14]. Traditional dynamic SLAM mitigates dynamic object interference through geometric constraints or auxiliary sensors. Recent advances in deep learning enable dynamic SLAM systems incorporating optical flow, depth estimation, and semantic segmentation techniques to achieve superior recognition accuracy. Motion segmentation-based methods [15], typically performing pixel-level image segmentation, impose significant computational overhead. Meanwhile, target detection-based approaches [16] that identify regions frequently containing background elements compromise dynamic point recognition accuracy, potentially inducing system localization failures.

To address these challenges, we propose OS-SLAM, a dynamic environment SLAM method employing optical flow motion segmentation. The framework integrates optical flow motion segmentation with lightweight instance segmentation to mitigate dynamic interference during visual odometry and mapping processes. This dual approach effectively eliminates dynamic perturbations, enabling the construction of static dense point cloud maps. The principal contributions of this work are four-fold:

(1) This study introduces OS-SLAM, a dynamic environment SLAM system that utilizes optical flow motion segmentation. The system combines motion and instance segmentation, enabling the accurate segmentation of non-rigid dynamic objects and the reconstruction of dense static scenes.
(2) To tackle challenges related to complex feature extraction of dynamic objects and imprecise long-distance motion estimation, we introduce a multi-scale optical flow network framework. This framework comprises a multi-scale feature extraction module and a multi-scale adaptive update module. Our approach aims to enhance the precision of estimating moving objects over long distances while ensuring computational efficiency.
(3) In order to mitigate the influence of non-rigid motion on segmentation precision, we present a segmentation framework that incorporates motion semantics. This framework includes a feature pyramid aggregator and a separable dynamic decoder for panoramic kernel generation. Additionally, it employs multi-head cross attention via separable dynamic convolution to effectively differentiate non-rigid moving objects from stationary backgrounds. This approach enhances the resilience of the SLAM system in dynamic settings.

The rest of this paper is organized as follows: Section 2 explores the technical ideas of existing deep learning dynamic SLAM, optical flow-based dynamic SLAM, and semantic segmentation-based dynamic SLAM solutions in depth, describing their methodological advantages as well as the limitations of existing methods in terms of cost and real-time performance. Section 3 describes our approach to developing a dynamic environment SLAM system based on optical flow motion segmentation. Section 4 describes our experimental results, demonstrating in detail the improvement effect of the optical flow part and comparing the performance indicators in dynamic scenes. Finally, Section 5 summarizes our contributions.

## 2. Related Work

### 2.1. Dynamic SLAM System Based on Deep Learning

Recent advances in deep neural networks have substantially enhanced robots semantic understanding capabilities of their environments. DS-SLAM [17] integrates semantic segmentation with motion consistency verification to mitigate dynamic object interference on localization accuracy while constructing dense semantic octree maps. DynaSLAM [18] extends ORB-SLAM2 by integrating Mask R-CNN for image semantic segmentation with multi-view geometry to detect dynamic objects. GAT-LSTM [19] uses a graph attention network (GAT) integrated into a long short-term memory (LSTM) network to enable the system to prioritize these stable feature points. The GAT component extracts spatial structural information from individual image feature points to model the local relationship of each FP. At the same time, the LSTM module facilitates the analysis of consistent temporal features of local associations. Blitz-SLAM [20] uses semantic information to help the SLAM system eliminate interference caused by moving objects. The original mask and the depth information of the moving object are combined to obtain a depth mask. The modified mask obtained by integrating the depth mask with the original mask can effectively cover the area of the moving object. At the same time, a method is proposed to determine whether a movable object has been in contact with a person, eliminating the phenomenon that the movable object appears in different locations on the global map. The image can be divided into an environmental region and a potential dynamic region by the bounding box of the moving object. The matching points in the environmental region are used to construct polarity constraints to eliminate outliers in the potential dynamic region.

### 2.2. Dynamic SLAM System Based on Optical Flow

The optical flow-based dynamic SLAM system performs dynamic filtering through optical flow residual analysis between consecutive image pairs. The feature-based STDyn-SLAM [21] system employs optical flow, SegNet, and depth maps for dynamic object detection. ORB features extracted from binocular images are used to compute optical flow between consecutive frames based on epipolar geometry. Points violating the epipolar constraints are classified as dynamic and subsequently rejected. The SP-FIOWSLAM [22] system replaces the ORB-SLAM2 original ORB feature extraction module with a self-supervised learning network SP-Flow for SLAM keypoint generation. TartanVO [23] represents a learning-based visual odometry framework that employs PWC-Net [24] as its matching network, demonstrating superior performance over geometry-based methods in challenging trajectory scenarios. The team proposed DytanVO [25] to optimize the forward-reasoning efficiency of TartanVO-based optical flow networks. RDMO-SLAM [26] enhances semantic integration by employing dense optical flow for the label augmentation and velocity estimation of map points through optical flow computation.

### 2.3. Dynamic SLAM System Based on Semantic Segmentation

Dynamic SLAM systems based on semantic segmentation integrate target detection models with traditional SLAM frameworks to identify and eliminate dynamic objects’ influence on feature points. These systems typically utilize semantic segmentation networks to generate dynamic object masks, remove corresponding feature points, and employ geometric constraints to optimize camera pose estimation while mitigating dynamic interference. RDS-SLAM [27] extends ORB-SLAM3 by decoupling semantic segmentation from the tracking thread, where movement probabilities propagate semantic information from the semantic thread to the tracking thread. This framework simultaneously detects and removes tracking outliers through movement probability analysis while maintaining real-time performance in dynamic environments. MMS-SLAM [28] is used as a multimodal scheme to improve the segmentation accuracy of dynamic object edges by moving fuzzy compensation and fusing point cloud clustering and segmentation results. RLD-SLAM [29] detects skewed objects with excessive static background interference that cause tracking failures, and introduces IMU constraints to correct these errors. PLDS-SLAM [30] distinguishes between static and dynamic objects by applying geometric constraints for line feature matching and epipolar geometry for point features. It employs semantic segmentation as a prior for dynamic objects and uses Bayesian theory to eliminate dynamic points. Peng et al. [31] integrated an asymmetric non-local neural (ANN) network for semantic segmentation and a static dense point cloud mapping thread. Through an innovative combination of deep image clustering based on the weighted variable K-Means algorithm and multi-view geometric constraints, the system can identify unknown dynamic objects when semantic information is unreliable. Li et al. [32] proposed a semantic SLAM method that combines panoramic cameras with LiDAR fusion, combined with data enhancement and instance segmentation of front-view images, and used SuperPoint for feature extraction. Three-dimensional dynamic landmarks are projected onto the segmentation results to generate a dynamic-static mask, thereby eliminating feature points in dynamic areas.

## 3. Method

### 3.1. OS-SLAM Framework

**Figure 1:**
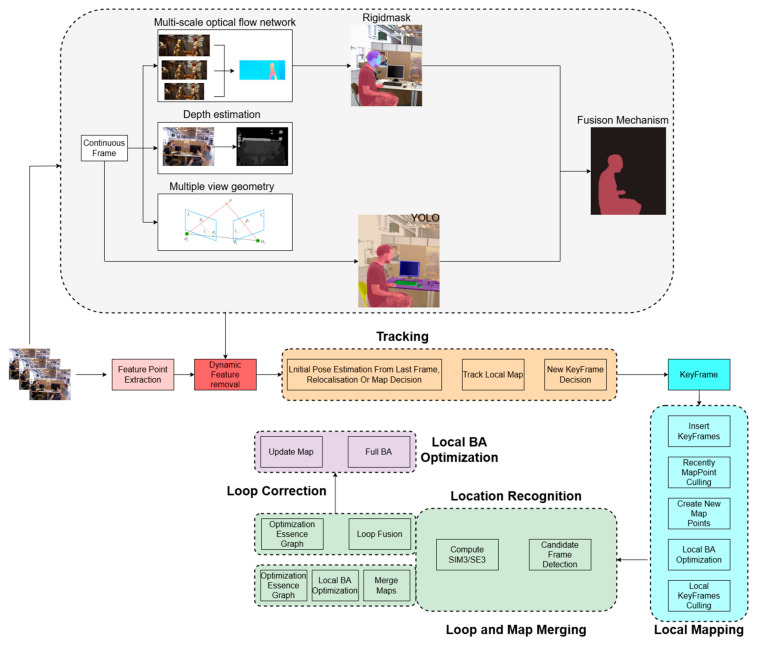
The framework of OS-SLAM.

Moving objects pose a significant challenge to SLAM systems, leading to a decline in both positioning accuracy and the integrity of map construction. This issue is mainly attributed to artifacts present in the static map representation, which are introduced during the processes of feature extraction and matching in SLAM. In pursuit of this objective, we have introduced an OS-SLAM system derived from ORB-SLAM3, designed to maintain stability in a stationary setting. The schematic representation of this process is depicted in Figure 1. Our approach involves the integration of the Rigidmask [33] and YOLO-fastest [34] post-motion segmentation frameworks into ORB-SLAM3 to enhance optical flow estimation networks. Specifically, Rigidmask is utilized for dynamic motion object estimation and segmentation in conjunction with YOLO-fastest prior to feeding the image into the tracking module. Within the motion segmentation module responsible for identifying moving objects, we have enhanced the optical flow estimation network by incorporating multi-scale refinements to enhance the accuracy of small object motion. Simultaneously, we have optimized the update module and loss function to address computational expenses associated with multi-scale processing, thereby achieving precise reconstruction of static point cloud maps.

In OS-SLAM, a fusion thread that integrates optical flow motion segmentation is incorporated into ORB-SLAM3, encompassing optical flow-based motion segmentation and semantic segmentation threads. The objective is to distinguish dynamic objects and preserve static feature points to enhance the accuracy of camera motion trajectory estimation. The optical flow thread supplies dynamic object features to Rigidmask for motion segmentation. To tackle optical flow estimation errors arising from non-rigid objects and residual dynamic features at segmentation boundaries, the outputs from both optical flow and semantic segmentation threads are combined. This fusion process helps mitigate disruptions caused by both rigid and non-rigid dynamic objects during tracking and mapping procedures.

### 3.2. Optical Flow Network Structure

**Figure 2:**
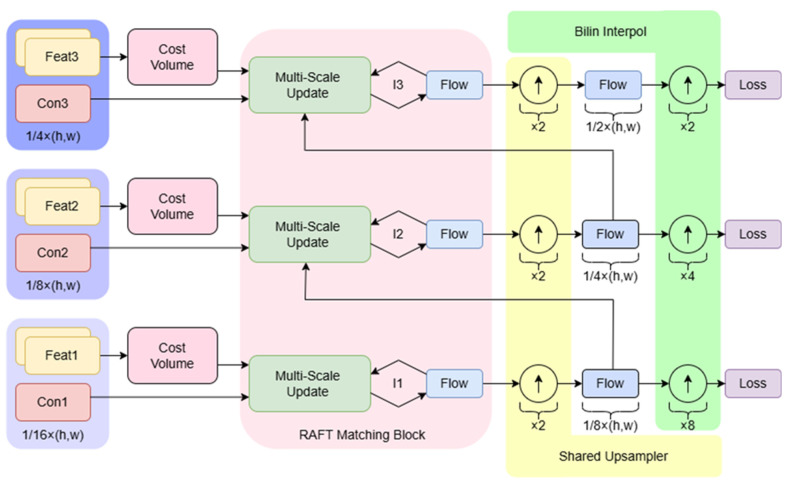
Multi-scale improved optical flow estimation network.

While PWC-Net has shown high accuracy in full-pixel prediction for optical flow estimation, its extensive computational requirements and inference delays hinder its real-time application. The network’s operations include multi-scale feature extraction, cost body construction, and multi-level optical flow prediction optimization. Particularly, constructing cost bodies and optimizing high-resolution layers involve intensive convolution and feature matching calculations, leading to high memory consumption and computational load. Additionally, Rigidmask’s performance heavily relies on precise input optical flow or scene flow. In scenarios with noise, errors, or inconsistencies in the motion field due to dynamic objects (common in complex scenes), its motion model fitting based on rigid assumptions may fail, resulting in inaccurate motion segmentation. To address this, we propose a lightweight optical flow estimation network inspired by RAFT [35]. Our solution implements coarse-to-fine feature extraction to mitigate feature information loss for moving objects across resolutions, while the redesigned update module reduces multi-scale computational costs. The architecture is illustrated in Figure 2.

The multiscale features are derived from two frames of image features (yellow) and contextual features from the initial frame (pink). Beginning at the coarsest scale, an image feature-based cost volume is generated, and a correlation pyramid is constructed following the RAFT framework. Correlation candidates are selected from the pyramid, and contextual features along with current motion estimates are collectively processed through a multi-scale update module. The refinement process commences at finer scales utilizing ×2 convex upsampling masks, with the iteration progressing through different scales. The ultimate outputs are interpolated to align with the original resolution.

#### 3.2.1. Multi-Scale Feature Extractor

**Figure 3:**
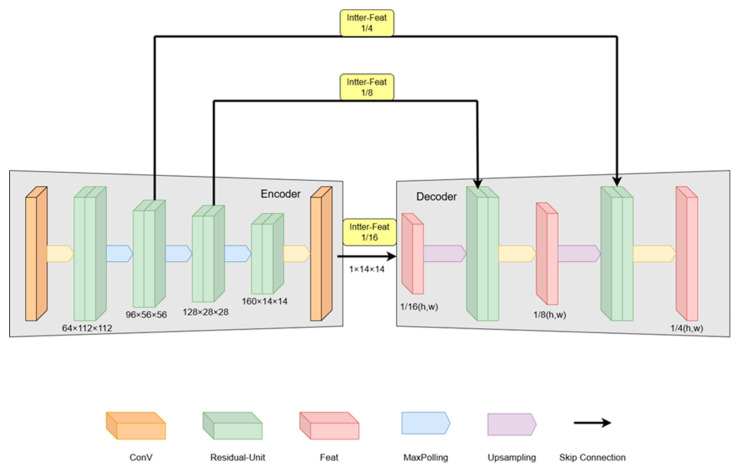
Multi-scale feature extractor.

Multi-scale image input and feature extraction are implemented by integrating ResNet residual units into the original RAFT encoder structure, following a U-Net-like architecture. This approach preserves hierarchical feature representation while enhancing the encoder’s capacity through residual connections. As shown in Figure 3, the input RGB frame is initially converted from 3 to 64 channels, then progressively downsampled via residual units to generate multi-scale intermediate features at resolutions of $\frac{1}{4}(h,w)$, $\frac{1}{8}(h,w)$, and $\frac{1}{16}(h,w)$. These features are fed into the enhancement module, where upsampling begins from the deepest $\frac{1}{16}(h,w)$ features to maintain dimensional consistency during residual summation. This hierarchical design ensures feature alignment across scales while preserving spatial coherence. Finally, the intermediate features (inter-Feat) from the current shallower scale are fused with upsampled deeper features through residual units. This fused output undergoes iterative upsampling and residual summation to propagate features toward shallower scales, thereby preserving deeper-scale features (Feat) throughout the iteration process. The multiscale contextual features share the same feature extractor structure as the base framework.

#### 3.2.2. Multi-Scale Update Module

**Figure 4:**
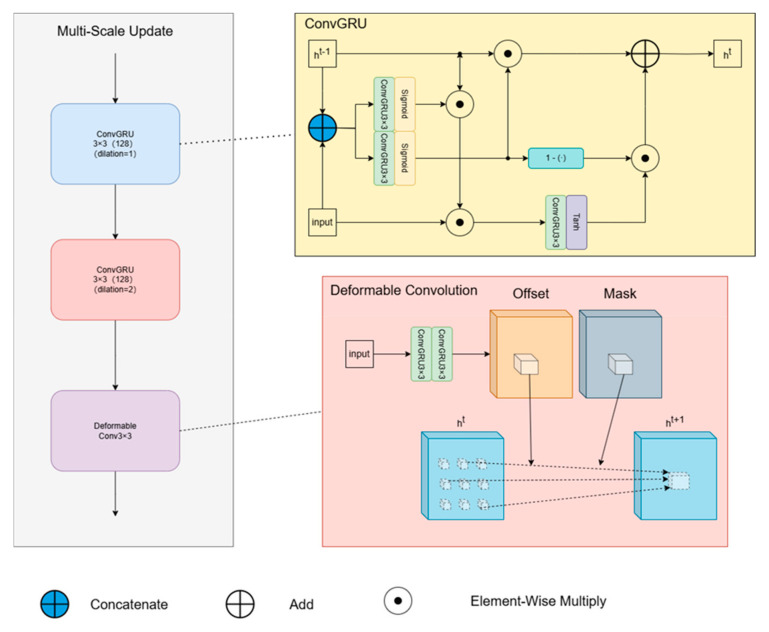
Multi-scale update module.

To tackle the challenge of optical flow estimation for dynamic objects exhibiting significant displacements, we introduce a novel multiscale update module that combines ConvGRU and deformable convolution (DCN). This framework facilitates the propagation of motion features across different spatial scales, ensuring coherence in displacement prediction. The initial segment of the module comprises a conventional ConvGRU alongside an augmented ConvGRU with a dilation rate of 2. In contrast to ConvLSTM, the ConvGRU framework employs a singular gated recurrent unit for computation, maintaining a streamlined design while enlarging the receptive field to handle substantial displacement complexities. The latter segment leverages deformable convolution to acquire knowledge of adaptive pixel offsets, as depicted in Figure 4.

We still use the original notation of RAFT to illustrate that the optical flow information processed by the multi-scale update module can be expressed as:

(1) $$z_{t}^{\left(\theta \right)}=\sigma (Conv_{3*3}^{D_{\theta }}([h_{t}^{\left(\theta -1\right)},x_{t}],W_{z}^{\left(\theta \right)}))$$

(2) $$r_{t}^{\left(\theta \right)}=\sigma (Conv_{3*3}^{D_{\theta }}([h_{t}^{\left(\theta -1\right)},x_{t}],W_{r}^{\left(\theta \right)}))$$

(3) $${\tilde{h_{t}}}^{\left(\theta \right)}=\tan h(Conv_{3*3}^{D_{\theta }}([r_{t}^{\left(\theta \right)}⊙h_{t}^{\left(\theta -1\right)},x_{t}],W_{\tilde{h}}^{\left(\theta \right)}))$$

(4) $$h_{t}^{\left(\theta \right)}=(1-z_{t}^{\left(\theta \right)})⊙h_{t}^{\left(\theta -1\right)}+z_{t}^{\left(\theta \right)}⊙{\overset{\sim }{h}}_{t}^{\left(\theta \right)}$$

(5) $$h_{t}^{\left(3\right)}(x)=\sum_{\chi \in N_{x}}K(\chi )\;*\;\chi h_{t}^{\left(2\right)}(x+\chi +P_{t}(x,\chi ))\;*\;M_{t}(x,\chi )$$

The equations describe the ConvGRU operations, where θ∈{1,2,3} denotes the layer index, and $h_{t}^{\left(\theta \right)}$ represents the hidden state at time step t. The input $x_{t}$ is the concatenation of previously defined features, namely optical flow, correlation, and context features. ⊙ denotes element-wise multiplication, while σ and tanh are the sigmoid and hyperbolic tangent activation functions, respectively. $D_{\theta }$ signifies the dilation rate for the convolutional operations within the ConvGRU. To enhance the capture of multi-scale motion information, the dilation rates for the two ConvGRUs are specifically set to 1 and 2. For the Deformable Convolutional Network component, its processing involves predicting P pixel offsets. These offsets are derived from both the input features of the update module and a set of M modulation masks (one per kernel weight). The operation aggregates features over a local neighborhood $N_{x}$, defined as the central pixel and its 8 immediate neighbors. K represents the spatially invariant convolution kernel weights.

#### 3.2.3. Multi-Scale Loss Function

Most current deep learning-based optical flow algorithms rely on supervised training, where models predict dense optical flow fields between consecutive image frames by using ground-truth optical flow as the supervisory signal. The training process commonly involves loss functions such as Mean Squared Error (MSE) and Endpoint Error (EPE). While MSE is simple to compute, its susceptibility to outliers can lead to noise sensitivity due to the absence of explicit spatial smoothness constraints. On the other hand, EPE, which calculates the L2 norm between predicted and ground-truth flow vectors per pixel, offers a more meaningful geometric evaluation. Nevertheless, computing EPE typically involves higher costs compared to MSE.

This paper proposes a multi-scale iterative loss function suitable for the algorithm in this paper by combining the multi-scale ideas of PWCNet and FlowNet and the advantages of MSE and EPE. Let $N_{rs}$ be the number of scales, $N_{iter(s)}$ be the number of iterations at that scale, and the overall loss is:

(6) $$L\left(\theta \right)=\sum_{s=1}^{N_{rs}}\sum_{i=1}^{N_{iter}\left(s\right)}\gamma_{s,i}\;L_{s,j}\left(\theta \right)$$

Let θ denote the set of all learnable parameters in the final network, encompassing both the feature pyramid extractor and the optical flow estimators (including upsampling and cost layers) at each pyramid level. Here, $N_{sample}$ represents the batch size, $N_{px}$ denotes the number of pixels per image, $f_{p}^{s,j}$ is the predicted optical flow vector at pyramid level s and iteration i for pixel, and $f_{p}^{gt}$ is the corresponding ground-truth flow vector.

(7) $$L_{s,i}\left(\theta \right)=\frac{1}{N_{sample}}\sum_{s=1}^{N}\frac{1}{N_{p}}\sum_{n=1}^{N_{p}}{\left|f_{p}^{s,i}-f_{p}^{gt}\right|}_{2}+\gamma {\left|\theta \right|}_{2}$$

where ${\left|\cdot \right|}_{2}$ computes the *L*2 norm of the vector and the second term regularizes the mode parameters. For fine-tuning, the following robust training loss is used.

(8) $$L_{s,i}^{final}\left(\theta \right)=\frac{1}{N_{sample}}\sum_{n=1}^{N_{sample}}{\left(\frac{1}{N_{px}}\sum_{m=1}^{N_{px}}\left|f_{p}^{s,i}-f_{p}^{gt}\right|+\varepsilon \right)}^{q}+\gamma {\left|\theta \right|}_{2}$$

where $\left|\cdot \right|$ is the *L*1 norm, and *q* < 1 (*q* = 0.7) has a smaller penalty on outliers, *θ* = 0.01.

### 3.3. Fusion Mechanism

**Figure 5:**
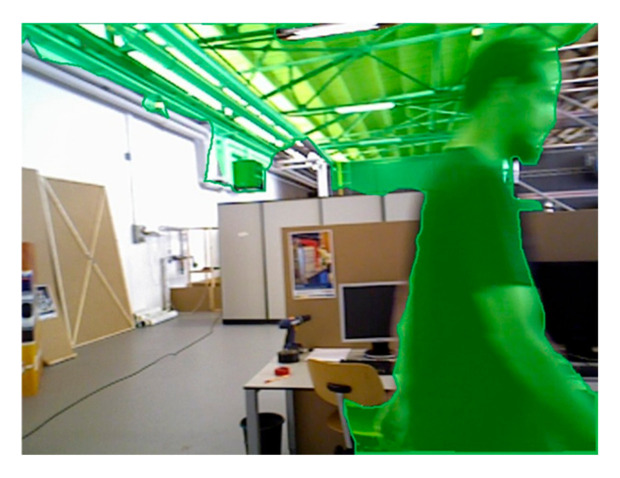
Static background segmentation error caused by non-rigid moving objects.

The Rigidmask framework utilizes segmentation mask prediction and 3D rigid transformation to parameterize background and multiple rigid moving objects. Nonetheless, segmentation errors may occur in static backgrounds when non-rigid motion components are present in the scene (see Figure 5). To address this issue, we introduce the YOLO-fastest real-time segmentation framework to enhance background segmentation accuracy in the presence of non-rigid motion. Experimental results demonstrate that the joint Rigidmask-YOLO segmentation approach outperforms the standalone Rigidmask segmentation method in accurately segmenting non-rigid moving objects and static backgrounds.

To enhance the fusion of Rigidmask and YOLO-fastest segmentation outcomes, we introduce a motion segmentation module that integrates Rigidmask-produced moving object masks with YOLO-fastest-derived semantic segmentation masks. This integration process aims to align and merge the outputs of these two models to enhance the accuracy of motion segmentation. The module analyzes a sequence of two consecutive images to forecast the center of the moving object using Rigidmask-generated masks. It utilizes a breadth-first search (BFS) approach to assess whether the overlap ratio of the YOLO-fastest model mask at the nearest marker point to the predicted center surpasses 0.9. Upon meeting this criterion, the YOLO-fastest mask supersedes the initial outcome as the ultimate output. The algorithmic framework is outlined as follows.

The Fusion Mechanism is summarized as Algorithm 1.
**Algorithm 1**: Joint Segmentation Fusion**Input:** $M_{R}$ //Rigidmask segment mask $M_{Y}$ // YOLO-fastest segment mask $\left[(I_{1},I_{2}),\ldots ,(I_{n-1},I_{n})\right]$ //Image sequence **Output:**
$\left[Mask_{1},Mask_{2},\ldots ,Mask_{n}\right]$ //moving object mask
1: **Initialize:** i=2, l=12
2: **While** i≤n **do** 3: $\left[MR_{i1},MR_{i2},\ldots ,MR_{ia}],[OR_{i1},OR_{i2},\ldots ,OR_{ia}\right]$ //Rigidmask masks and predicts objects 4:  $\left[MR_{i1},MR_{i2},\ldots ,MR_{ib}\right]$ // YOLO-fastest masks 5:  **for**
j≤a **do** 6:   **for**
k≤b **do** 7:    q=queue(),q.Enqueue(p) //object prediction center point p 8:    **While**
q≠null **do** 9:      $p_{i}=q.Dequeue()$//mask marker closest to the center point access $p_{i}$ 10:     **If** points marked by the mask ** return** $p_{i}$ 11:     **else** 12:      **for** adjacent point that has not been accessed** do** 13:       q.Enqueue(); 14:     **end** 15:   **end** 16:   **If**
$p_{i}\in MY_{ik}∥R_{k}>0.9$ **then** //inclusion rate $R_{k}=\frac{\left|MR_{ik}\cup MY_{ik}\right|}{\left|MY_{ik}\right|}$ 17:      **for** adjacent point that has not been accessed **do** 18:       q.Enqueue(); 19:      $Mask_{i}=MY_{ik};$ 20:      l+=1; 21:   **end** 22:  **end** 23: **end** 24: **end** 25: **return**
$\left[Mask_{1},Mask_{2},\ldots ,Mask_{n}\right]$


### 3.4. Mapping

ORB-SLAM3 primarily relies on spatial geometric data for mapping, showing limitations in handling dynamic objects. The simultaneous segmentation of repetitive images with dynamic residual features results in a decline in mapping accuracy, where these point clouds are identified as abnormal noise artifacts. This is due to the fact that the positions of moving objects exhibit changes between frames, leading to points that appear stationary across consecutive frames. Additionally, point clouds at the edges of moving objects in segmentation outcomes exhibit sparser distributions compared to stationary objects. To address these issues, this study enhances ORB-SLAM3 by incorporating static dense point cloud mapping processes. Sparse dynamic point clouds are processed using SOR filtering, and moving objects are predicted using KD-tree-based centroid estimation, followed by statistical analysis. The average distances between points and their neighbors are computed from KD-tree results to characterize dynamic attributes. By assuming a Gaussian distribution for these distances, points with average distances outside the standard range are identified as outliers and subsequently excluded from the dataset.

## 4. Experiment and Results

### 4.1. Optical Flow Dataset Description and Training Strategy

The KITTI (Karlsruhe Institute of Technology and Toyota Technological Institute) [36] dataset is a street scene dataset captured in real-world traffic environments. It comprises two distinct subsets: KITTI 2012 and KITTI 2015. The dataset provides a total of 394 training image pairs and 395 test image pairs. The KITTI 2012 test set features static scenes, while the KITTI 2015 test set incorporates dynamic backgrounds. Data acquisition involves a LiDAR scanner operating at 10 frames per second (FPS), capturing approximately 100,000 points per scan cycle. Synchronized cameras are triggered at 10 FPS. Individual images within the dataset may contain up to 15 vehicles and 30 pedestrians.

The MPI-Sintel (Max Planck Institute Sintel) [37] optical flow dataset is a synthetic benchmark derived from an animated film. It comprises two distinct subsets: Clean and Final. The Clean subset contains challenging scenarios including large displacements, textureless regions, and significant non-rigid deformations. The Final subset further enhances realism by incorporating motion blur, atmospheric effects (fog), and image noise on top of the Clean imagery, thereby increasing the complexity of optical flow estimation. The dataset provides 1041 training image pairs and 552 test image pairs.

To validate the effectiveness of the proposed algorithm, it was implemented in Python and evaluated under the Ubuntu 20.04 operating system. Experiments were conducted on a platform equipped with an Intel i5-12400F CPU@4.4 GHz processor and accelerated using an NVIDIA RTX 3090 GPU (24GB). Adopting the training strategy and leveraging the initial weights from the original RAFT model, the model was first pre-trained on the FlyingThings dataset [38] for 100,000 iterations with a batch size of 12. Subsequently, it was trained on the FlyingThings3D dataset [39] for 100,000 iterations using a batch size of six. Following a methodology similar to MaskFlowNet [40] and PWCNet+, we then combined the MPI-Sintel, KITTI-2015, and HD1K [41] datasets and performed fine-tuning specifically on the MPI-Sintel dataset for an additional 100,000 iterations. Finally, the model weights obtained from the MPI-Sintel fine-tuning stage were used to initialize further fine-tuning on the KITTI-2015 dataset for 50,000 iterations.

### 4.2. Optical Flow Experiment Evaluation Criteria

On the KITTI-2015 dataset, optical flow estimation performance is evaluated using two primary metrics: the Endpoint Error (EPE), a standard error measure in optical flow estimation, and the percentage of optical flow outliers (Fl). The Endpoint Error quantifies the average Euclidean distance between the ground-truth optical flow vectors and the predicted vectors across all pixels. Its mathematical formulation is presented in Equation (9).

(9) $$EPE=\sqrt{\sum_{i=1}^{n}{\left(F_{i}-F_{gi}\right)}^{2}}$$

where $F_{i}$ and $F_{gi}$ denote the predicted optical flow vector and the corresponding ground-truth vector at pixel i, respectively. The Fl metric represents the ratio of optical flow outliers across the entire image region within the KITTI-2015 benchmark. It is defined as the proportion of pixels where the estimated optical flow error exceeds a specified threshold. A pixel is classified as an outlier if its estimated flow vector $\hat{u}$ and ground-truth vector $u_{gt}$ satisfy the following condition.

(10) $$\left\{\begin{array}{l} {∥u_{gt}-\hat{u}∥}_{2}>3\\ {∥u_{gt}-\hat{u}∥}_{2}/{∥u_{gt}∥}_{2}>0.05 \end{array}\right.$$

On the MPI-Sintel dataset, EPE and 1, 3, and 5 px are used as performance metrics, where 1, 3, and 5 px represent the proportion of pixels with EPE < 1, EPE < 3, and EPE < 5 in the entire image, respectively.

### 4.3. Optical Flow Comparison Experiment

To benchmark the improved RAFT against the original model, comparative experiments were conducted following the evaluation standards of the MPI-Sintel and KITTI-2015 datasets. Quantitative results are presented in Table 1, which compare our method against seven established optical flow algorithms: FlowNet, PWC-Net+, SCV, RAFT, RFPM, and GMA.

The experimental results are shown in Table 1, where EPE represents the average Endpoint Error of all pixels, Matched represents the Endpoint Error of the visible area in adjacent frames, Unmatched represents the Endpoint Error of the visible area only in one of the adjacent frames, F1-all represents the average percentage of optical flow outliers in the image, and F1-noc represents the average percentage of outliers in the occluded area in the image. The improved method has good generalization ability on both the Clean and Final datasets of MPI-Sintel, and is always better than RAFT, with an accuracy improvement of about 13.1%. At the same time, it is compared with the mainstream optical flow estimation models in recent years, and the best results are indicated in bold.

**Figure 6:**
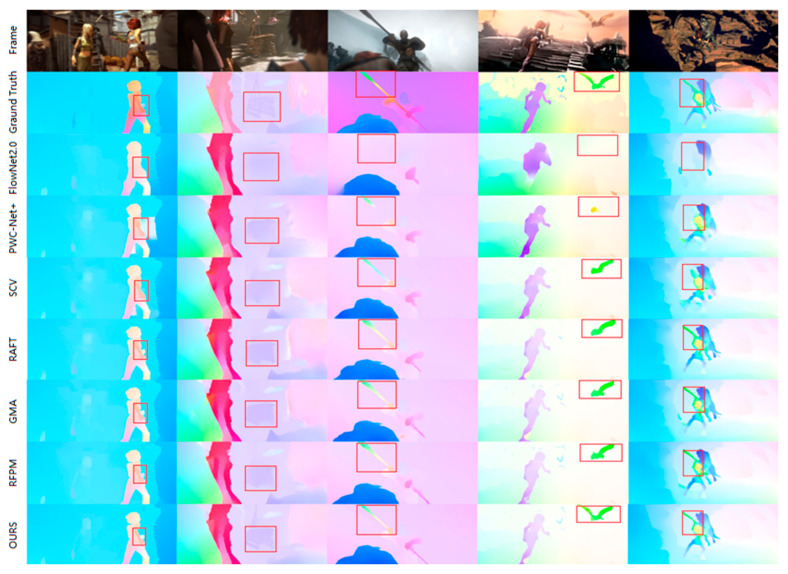
Compare experimental visualizations on the MPI-Sintel test set.

On the MPI-Sintel benchmark, the proposed method shows superior optical flow estimation accuracy on the Sintel Clean channel compared with mainstream networks including FlowNet2, SCV, Flow, GMA, and RFPM. It also achieves quite good performance on the more challenging Sintel Final channel. However, the proposed method is slightly inferior to GMA in terms of the Unmatched metric. This is attributed to the global aggregation module of GMA, which propagates high-confidence information to occluded areas using high-level features and global motion priors. In contrast, the proposed method solves the occlusion problem by fusing richer multi-scale feature details, but our method handles edge features of multi-scale changing objects better. As shown in Figure 6, which shows the MPI-Sintel optical flow estimation results, the proposed method can maintain robust estimation performance even for large displacement, fast-moving edge features such as those in the stick, bird, and beast sequences (the last three sequences), and the optical flow estimation of edge details of dynamic objects is significantly better than GMA. This capability mainly stems from the novel multi-scale framework and feature extractor introduced in this paper. These components effectively capture multi-scale information and comprehensively utilize the contextual features of each scale, thereby significantly improving the accuracy of optical flow estimation and improving feature matching at different scales.

**Figure 7:**
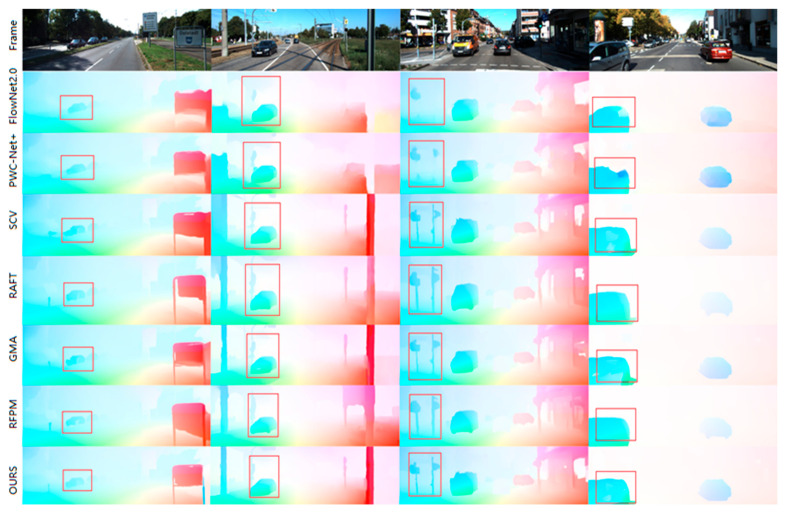
Experimental visualizations were compared on the KITTI-2015 test set.

On the KITTI benchmark, as presented in Table 1, the proposed method also achieves state-of-the-art performance in terms of both accuracy and robustness for optical flow estimation in real-world driving scenes. Visualization results comparing the proposed method with mainstream models on KITTI are provided in Figure 7. Analysis of key regions highlighted in the figure demonstrates the method’s significant effectiveness in estimating the optical flow for large-displacement objects, such as vehicles, and small targets, including telephone poles, streetlights, and billboards. Furthermore, it achieves optimal performance in occluded regions. A discernible discrepancy persists between the estimated optical flow accuracy at the edges of real-world objects on the road (e.g., curbs, lane markings) and the requirements for practical applica.

### 4.4. Ablation Experiment

Ablation studies are conducted to validate the effectiveness of the multi-scale framework proposed in this paper, specifically examining the contributions of the Res-UNet-based feature extractor, the multi-scale update module, and the multi-scale tailored loss function. Models are pre-trained on the FlyingChairs and FlyingThings3D datasets and evaluated on the MPI-Sintel benchmark, as shown in Table 2. Initially, we investigate the impact of increasing the feature pyramid depth within a single-scale RAFT framework from 4 to 5 layers under varying iteration counts (where 18 iterations correspond to the multi-scale refinement setting). Results indicate that this increase yields only marginal improvements in accuracy. Subsequently, considering the computational overhead of optical flow iteration and model parameter count, we evaluate accuracy across different pyramid layer configurations (2, 3, and 4 layers) based on scales 2 and 3. The configuration utilizing 2 scales and 3 layers demonstrates optimal evaluation accuracy. Finally, the individual contributions of the proposed feature extractor, update module, and loss function are verified. Replacing the corresponding modules in RAFT with our proposed components leads to significant improvements in evaluation accuracy.

### 4.5. SLAM Dataset Description

To evaluate the localization accuracy and mapping performance, we validated the method using the RGB-D dataset from the Technical University of Munich (TUM). This dataset uses Microsoft Kinect and Asus Xtion to provide RGB and depth images, and 8 high-speed infrared cameras to track the camera pose through markers with millimeter-level accuracy. The dataset contains 39 indoor sequences, including weakly textured scenes, dynamic objects, 3D reconstruction, and calibration files such as camera intrinsics, which supports comprehensive performance evaluation across multiple tasks. To evaluate the robustness of the SLAM algorithm to dynamic objects, we use a dynamic object dataset containing fr3_sitting_xyz (slow movement) and fr3_walking_xyz (fast movement) sequences. Trajectory evaluation is performed using TUM’s evo trajectory accuracy evaluation tool.

### 4.6. Error Evaluation

To evaluate the proposed algorithm’s performance, we employ two metrics to assess SLAM trajectory global coherence: Absolute Position Error (APE) and Relative Position Error (RPE).

APE is the absolute attitude error between two attitudes $P_{i}$ and $Q_{i}$ at timestamp i, where $P_{i}$ is the estimated pose and $Q_{i}$ is the true pose. APE is expressed as:

(11) $$APE=\sqrt{\left(\frac{1}{n}{\sum_{i=1}^{n}\left\|trans(Q_{i}^{-1}P_{i})\right\|}^{2}\right)}$$

RPE is the relative attitude error between two attitudes $P_{i}$ and $Q_{i}$ at timestamp i, where $P_{i}$ is the estimated pose and $Q_{i}$ is the true pose. RPE is expressed as:

(12) $$RPE=\sqrt{\left(\frac{1}{M}{\sum_{i=1}^{m}\left\|trans((Q_{i}^{-1}Q_{i}+△t)(P_{i}^{-1}P_{i}+△t))\right\|}^{2}\right)}$$

### 4.7. Experimental Comparison and Analysis

**Figure 8:**
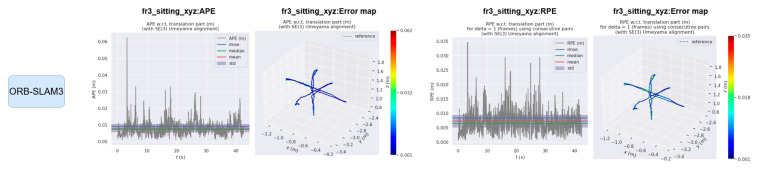
APE and RPE of ORB-SLAM3 in fr3_sitting_xyz.

**Figure 9:**
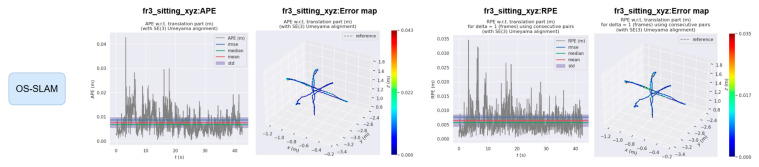
APE and RPE of OS-SLAM in fr3_sitting_xyz.

**Figure 10:**
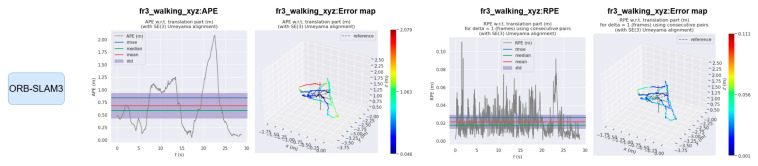
APE and RPE of ORB-SLAM3 in fr3_walking_xyz.

**Figure 11:**
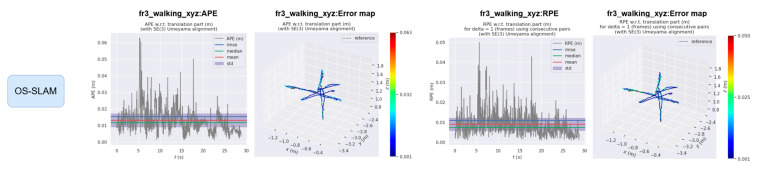
APE and RPE of OS-SLAM in fr3_walking_xyz.

The EVO evaluation tool was used to compare the error between the proposed method and ORB-SLAM3. Figure 8 and Figure 9 compare the absolute pose error (APE) and relative pose error (RPE) of ORB-SLAM3 and the proposed system OS-SLAM under fr3_sitting_xyz. Figure 10 and Figure 11 compare the APE and RPE of ORB-SLAM3 and the proposed system OS-SLAM under fr3_walking_xyz.

**Figure 12:**
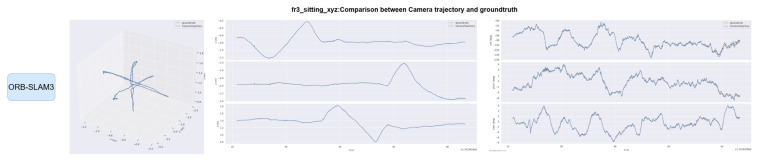
Comparison of ORB-SLAM3 camera trajectory on the fr3_sitting_xyz sequence with the true trajectory.

**Figure 13:**
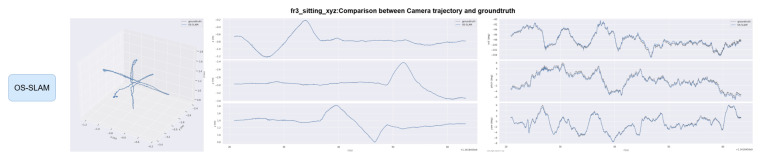
Comparison of OS-SLAM camera trajectory on the fr3_sitting_xyz sequence with the true trajectory.

**Figure 14:**
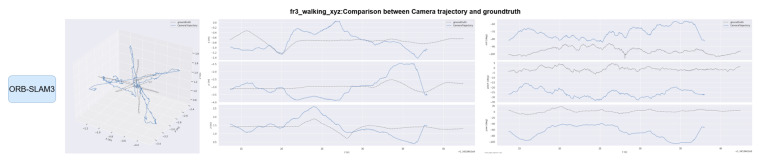
Comparison of ORB-SLAM3 camera trajectory on the fr3_walking_xyz sequence with the true trajectory.

**Figure 15:**
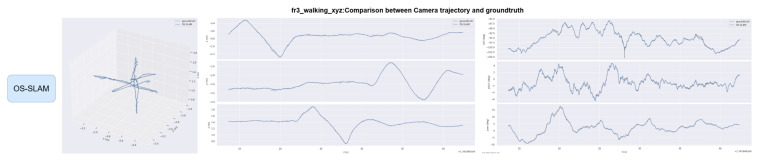
Comparison of OS-SLAM camera trajectory on the fr3_walking_xyz sequence with the true trajectory.

Trajectories of OS-SLAM and ORB-SLAM3 are compared on both sequences. Figure 12 and Figure 13 compare the trajectory curves of the two methods in the fr3_sitting_xyz sequence, and Figure 14 and Figure 15 compare the trajectory curves of the two methods in the fr3_walking_xyz sequence, and provide axis alignment comparisons of translation (xyz) and rotation (pitch, yaw, roll) parameters in 3D space.

Error and trajectory curve comparisons demonstrate that ORB-SLAM3 achieves stable performance in slow dynamic sequences, but exhibits significant tracking accuracy degradation in fast dynamic scenarios. In contrast, our method maintains consistent accuracy across both slow and fast dynamic environments through dynamic target segmentation.

Table 3 analysis reveals a 0.27% difference in absolute position APE between ORB-SLAM3 and our method during slow dynamic sequences, with our approach showing marginally superior performance. In fast dynamic scenarios, our method achieves 38.78% lower RMSE compared to ORB-SLAM3, demonstrating significant tracking accuracy improvement in dynamic environments. Table 4 demonstrates comparable RPE values between OS-SLAM and ORB-SLAM3 in slow dynamic sequences under both translational and rotational conditions. In fast dynamic scenarios, our method reduces translational relative pose error RMSE by 51.57% and rotational relative pose error RMSE by 32.87% compared to ORB-SLAM3. These results confirm the superior accuracy of our approach in dynamic environments for both absolute and relative positional metrics.

To comprehensively evaluate OS-SLAM performance in dynamic environments, three challenging sensor trajectory sequences from the TUM dataset were tested: “fr3_walking_static”, “fr3_walking_rpy”, and “fr3_walking_halfsphere”. Table 5 and Table 6 present APE and RPE pose error comparisons between two sequences, respectively. Experimental comparisons with current mainstream SLAM systems demonstrate OS-SLAM superior accuracy in dynamic environments.

To evaluate computational efficiency, tracking and segmentation performance metrics along with CPU/GPU utilization are compared with mainstream dynamic SLAM systems, as shown in Table 7**.** While OS-SLAM shows limited segmentation time improvement due to optical flow-based motion segmentation, its optimized optical flow network inference and YOLO lightweight architecture achieve superior hardware efficiency compared to YOLO-based SLAM systems.

**Figure 16:**
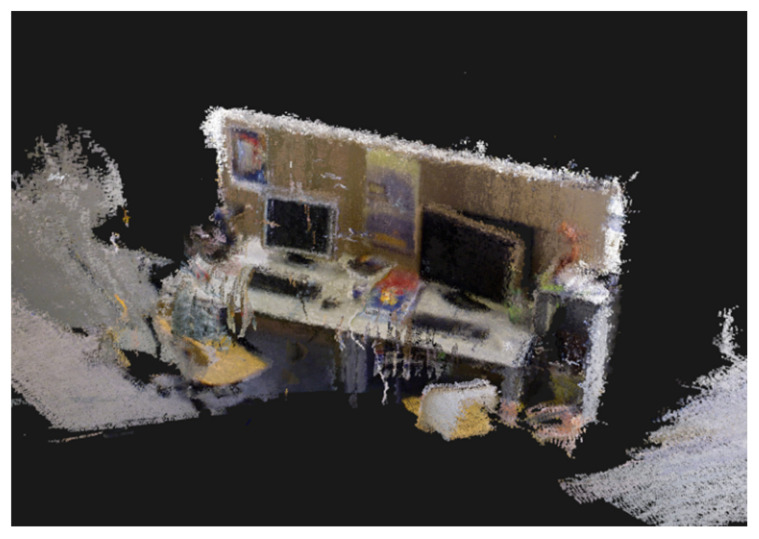
Dense point cloud image after dynamic segmentation by OS-SLAM.

To enhance practical applicability, we implement dense point cloud mapping and evaluate OS-SLAM performance using the fast dynamic sequence fr3_walking_xyz. Figure 16 demonstrates the reconstruction results, where joint motion segmentation and instance segmentation eliminate dynamic objects while reducing static background reconstruction errors. Residual dynamic contour point clouds are further processed through anomaly filtering, enabling the resulting maps to accurately represent static environments for navigation and localization tasks.

## 5. Conclusions

OS-SLAM is an innovative real-time dynamic Simultaneous Localization and Mapping (SLAM) algorithm tailored for indoor environments devoid of Global Navigation Satellite System (GNSS) support. This algorithm integrates optical flow motion segmentation with instance segmentation to effectively detect and segment dynamic objects. By enhancing the multi-scale architecture of the optical flow network and optimizing its update module, the algorithm enhances the accuracy of dynamic object estimation while maintaining a lightweight network structure. To address errors stemming from mis-segmentation of the static background due to non-rigid moving objects, OS-SLAM is coupled with the YOLO-fastest algorithm. Subsequently, Sparse Outlier Removal (SOR) filtering is employed on the sparse point cloud featuring dynamic attributes to eliminate outliers. Furthermore, a dedicated dense mapping thread is incorporated to generate dense point cloud maps devoid of dynamic objects. The performance of OS-SLAM is assessed using the TUM RGB-D dataset. Ablation experiments demonstrate substantial enhancements in the performance of each module within the designed optical flow network. Comparative analysis with ORB-SLAM3 reveals that OS-SLAM achieves a notable average reduction of 91.2% in Absolute Position Error (APE) and 44.1% in Relative Position Error (RPE) in high dynamic sequences. Additionally, OS-SLAM exhibits average tracking and segmentation times below 40ms and 20ms, respectively, outperforming mainstream dynamic SLAM systems. Moreover, OS-SLAM demonstrates notable advantages in terms of CPU and GPU utilization.

## Figures and Tables

**Table 1:** Results on Sintel (test) and KITTI (test).

Method	Sintel Clean (Test)			Sintel Final (Test)			KITTI (Test)	
	All	Mat	Unmat	All	Mat	Unmat	F1-All	Fl-Noc
FlowNet2.0	1.92	0.91	12.59	3.84	1.81	24.00	11.48	-
PWC-Net+	1.71	0.55	11.07	3.45	1.65	17.40	7.72	-
SCV [42]	1.72	0.57	11.08	3.60	1.70	19.14	6.17	-
RAFT [43]	1.61	0.62	9.65	2.86	1.41	14.68	5.10	3.07
GMA [44]	1.39	0.58	7.96	2.47	1.24	**12.50**	4.93	2.90
RFPM [45]	1.41	**0.49**	8.88	2.90	1.33	15.69	**4.79**	2.85
OURS	**1.40**	0.52	**8.75**	**2.66**	**1.23**	14.70	4.92	**2.82**
Improvement over RAFT	+13.1%	+16.1%	+9.3%	+6.9%	+12.7%	−0.1%	+3.5%	+8.1%

**Table 2:** Ablation study.

Pre-Trained on Chairs and Things	Sintel (Train)	
	Clean	Final
**1. Single-Scale RAFT:** Finest Scale/Recurrent Iterations		
1/8x(h,w), 12 iter (RAFT)	1.40	2.67
1/8x(h,w), 18 iter	1.45	2.70
1/4x(h,w), 12 iter	1.58	3.10
1/4x(h,w), 18 iter	1.52	3.08
**2. Multi-Scale RAFT:** Resolution Scales/Look-Up Levels		
2 scales/3 levels	1.16	3.07
2 scales/4 levels	1.14	2.64
2 scales/5 levels	1.11	2.66
3 scales/2 levels (ours)	1.13	2.60
3 scales/3 levels	1.15	2.66
3 scales/4 levels	1.14	2.70
**3. Update Module:** Multi-scale Update vs. Standard		
Multi-scale Update (ours)	1.12	2.61
Standard	1.22	2.67
**4. Multi-Scale Features:** U-Net-style vs. Standard		
U-Net-style (ours)	1.13	2.60
Standard	1.11	2.68
**5.Multi-Scale Loss:** Single-Scale vs. Multi-Scale		
Multi-scale loss (ours)	1.13	2.60
Single-scale loss	2.26	4.09

**Table 3:** Comparison of APE between the proposed method and ORB-SLAM3.

APE				
Error	ORB-SLAM3		OS-SLAM	
	fr3_sitting_xyz	fr3_walking_xyz	fr3_sitting_xyz	fr3_walking_xyz
Max	0.0467	0.9316	0.0833	0.0527
Mean	0.0141	0.3498	0.0125	0.0133
Median	0.0122	0.2235	0.0119	0.0117
Min	0.0014	0.0564	0.0013	0.0007
Rmse	0.0156	0.4089	0.0137	0.0151
Sse	0.3073	142.8564	0.2129	0.1863
Std	0.0064	0.2044	0.0055	0.0069

**Table 4:** Comparison of RPE between the proposed method and ORB-SLAM3.

RPE								
Error	ORB-SLAM3				OS-SLAM			
	fr3_sitting_xyz		fr3_walking_xyz		fr3_sitting_xyz		fr3_walking_xyz	
	T-P	R-P	T-P	R-P	T-P	R-P	T-P	R-P
Max	0.0377	0.0278	0.1894	0.1295	0.0637	0.0288	0.0551	0.1223
Mean	0.0068	0.0061	0.0169	0.0107	0.0077	0.0064	0.0095	0.0063
Median	0.0061	0.0053	0.0121	0.0072	0.0067	0.0054	0.0079	0.0052
Min	0.0006	0.0004	0.0008	0.0007	0.0002	0.0003	0.0007	0.0004
Rmse	0.0081	0.0074	0.0232	0.0139	0.0093	0.0077	0.0113	0.0092
Sse	0.0890	0.0735	0.4603	0.1660	0.1153	0.0787	0.1080	0.0798
Std	0.0043	0.0041	0.0155	0.0093	0.0049	0.0043	0.0059	0.0061

**Table 5:** RMSE analysis of APE.

Sequences	ORB-SLAM2	ORB-SLAM3	DynaSLAM	DS-SLAM	DM-SLAM	OS-SLAM
fr3_walking_xyz	0.7830	0.4019	0.0154	0.2460	0.0125	0.0133
fr3_walking_static	0.3851	0.0671	0.0063	0.0079	0.0139	0.0138
fr3_walking_halfsphere	0.4652	0.4228	0.0284	0.0297	0.0266	0.0272
fr3_walking_rpy	0.7831	0.6525	0.0341	0.4372	0.0376	0.0365

**Table 6:** RMSE analysis of RPE (Translational posture).

Sequences	ORB-SLAM2	ORB-SLAM3	DynaSLAM	DS-SLAM	DM-SLAM	OS-SLAM
fr3_walking_xyz	0.0423	0.0224	0.0205	0.0321	0.0233	0.0116
fr3_walking_static	0.0297	0.0207	0.0086	0.0105	0.0077	0.0042
fr3_walking_halfsphere	0.0483	0.0202	0.0361	0.0241	0.0319	0.0139
fr3_walking_rpy	0.1695	0.0273	0.0436	0.0461	0.0233	0.0217

**Table 7:** Times and calculated cost evaluation.

Method	Average Tracking Times (ms)	Average Segmentation Times (ms)	CPU	GPU
Normal state	/	/	3%	2%
ORB-SLAM3	>100	/	30%	3%
DynaSLAM	>100	192.00	61%	35%
DS-SLAM	67.30	55.15	53%	32%
YOLOv5-SLAM	79.52	47.63	40%	23%
YOLOv8-SLAM	51.66	22.32	44%	27%
OS-SLAM	35.32	18.10	37%	23%

## Data Availability

The data supporting the findings of this study are publicly available at the official websites: MPI-Sintel (http://sintel.is.tue.mpg.de/), KITTI-2015 (https://www.cvlibs.net/datasets/kitti/evalsceneflow.php?benchmark=stereo), and TUM (https://vision.in.tum.de/data/datasets/rgbd-dataset/download).

## Abbreviations

SLAM	Simultaneous Localization and Mapping
GNSS	Global Navigation Satellite System
OS-SLAM	Optical flow motion segmentation-based SLAM
VO	Visual Odometry
BA	Bundle Adjustment
AR	Augmented Reality
UAV	Unmanned Aerial Vehicle
IMU	Inertial Measurement Unit
ANN	Asymmetric Non-local Neural network
YOLO	You Only Look Once
DCN	Deformable Convolutional Network
ConvGRU	Convolutional Gated Recurrent Unit
BFS	Breadth-First Search
SOR	Statistical Outlier Removal
KD-Tree	K-Dimensional Tree
TUM	Technical University of Munich
KITTI	Karlsruhe Institute of Technology and Toyota Technological Institute
MPI-Sintel	Max Planck Institute Sintel
APE	Absolute Position Error
RPE	Relative Position Error
EPE	Endpoint Error
RMSE	Root Mean Square Error
SSE	Sum of Squared Errors
STD	Standard Deviation
ORB	Oriented FAST and Rotated BRIEF
